# A Single Nucleotide Change Affects Fur-Dependent Regulation of *sodB* in *H. pylori*

**DOI:** 10.1371/journal.pone.0005369

**Published:** 2009-04-28

**Authors:** Beth M. Carpenter, Hanan Gancz, Reyda P. Gonzalez-Nieves, Abby L. West, Jeannette M. Whitmire, Sarah L. J. Michel, D. Scott Merrell

**Affiliations:** 1 Department of Microbiology and Immunology, Uniformed Services University of the Health Sciences, Bethesda, Maryland, United States of America; 2 Department of Pharmaceutical Sciences, School of Pharmacy, University of Maryland, Baltimore, Maryland, United States of America; University of Hyderabad, India

## Abstract

*Helicobacter pylori* is a significant human pathogen that has adapted to survive the many stresses found within the gastric environment. Superoxide Dismutase (SodB) is an important factor that helps *H. pylori* combat oxidative stress. *sodB* was previously shown to be repressed by the Ferric Uptake Regulator (Fur) in the absence of iron (*apo*-Fur regulation) [1]. Herein, we show that *apo* regulation is not fully conserved among all strains of *H. pylori*. *apo*-Fur dependent changes in *sodB* expression are not observed under iron deplete conditions in *H. pylori* strains G27, HPAG1, or J99. However, Fur regulation of *pfr* and *amiE* occurs as expected. Comparative analysis of the Fur coding sequence between G27 and 26695 revealed a single amino acid difference, which was not responsible for the altered *sodB* regulation. Comparison of the *sodB* promoters from G27 and 26695 also revealed a single nucleotide difference within the predicted Fur binding site. Alteration of this nucleotide in G27 to that of 26695 restored *apo*-Fur dependent *sodB* regulation, indicating that a single base difference is at least partially responsible for the difference in *sodB* regulation observed among these *H. pylori* strains. Fur binding studies revealed that alteration of this single nucleotide in G27 increased the affinity of Fur for the *sodB* promoter. Additionally, the single base change in G27 enabled the *sodB* promoter to bind to *apo*-Fur with affinities similar to the 26695 *sodB* promoter. Taken together these data indicate that this nucleotide residue is important for direct *apo*-Fur binding to the *sodB* promoter.

## Introduction

*Helicobacter pylori* is an important human pathogen that infects over 50% of the world's population [2]. While infection is predominantly asymptomatic, this bacterium is associated with development of gastritis, peptic ulcer disease, mucosa-associated lymphoid tissue lymphoma, and gastric adenocarcinoma. Infection often occurs early in childhood and persists throughout a person's lifetime unless they are treated with specific antibiotics [3]. Given its propensity for chronic colonization and the substantial number of infected individuals, *H. pylori* imposes a significant disease burden worldwide.

This microaerophilic, Gram negative bacterium is interesting in that it colonizes and survives within the gastric mucosa of the human stomach. *H. pylori* is well suited to life within this niche and has many factors that enable it to thrive there [2], [4]. One such factor, the *F*erric *u*ptake *r*egulator (Fur), functions as a transcriptional regulator that is involved in maintaining iron homeostasis [5]. Iron is essential for bacterial survival and is a co-factor in a variety of proteins; however, iron is redox active and can promote oxidative damage making it imperative that intracellular iron levels are tightly controlled. One particularly deleterious reaction that free iron can promote is reaction with reactive oxygen species (ROS) to form highly reactive hydroxyl radicals via Fenton chemistry. Hydroxyl radicals cause DNA and cellular damage that eventually lead to cell death. Thus, cells must strive to maintain a balance between insufficient and excess iron. Fur is involved in preserving this fine balance in *H. pylori*, and consequently, it is not surprising that *fur* has been shown to be critical for colonization in both gerbil and murine models of infection [6], [7].

Fur is conserved in a wide variety of bacterial species and functions similarly in all of them by repressing gene expression under conditions of sufficient cellular iron. When Fur is bound to its iron (Fe^2+^) co-factor, it binds to specific regions in iron-regulated promoters called Fur Boxes and blocks the binding of RNA polymerase. Genes regulated in this manner are often associated with iron acquisition and are repressed under iron replete conditions to prevent the harmful effects of iron overload. While *H. pylori* Fur has been found to repress a set of genes in its iron-bound state, it has also uniquely been found to repress an additional set of genes in the absence of the iron cofactor, i.e. when Fur is in its *apo* form. *apo*-Fur regulation involves repression of an iron storage gene and occurs under iron limited conditions [8].

*apo*-Fur regulation has not been described for other bacterial species, and given that Fur plays a role in global gene regulation in response to environmental stressors and enhances the fitness of *H. pylori* as a pathogen, functional studies of Fur in *H. pylori* are of particular interest. One gene known to be repressed by *apo*-Fur in *H. pylori* that is not directly linked to iron metabolism, but is involved with the oxidative stress response, is *superoxide dismutase* (*sodB*) [1]. SodB was first identified in *H. pylori* in 1993 and was shown to be iron co-factored like the *Escherichia coli* FeSod with 53.5% identity between the two proteins [9]. However, unlike *E. coli* FeSod, which is localized within the cytosol of the bacterium, *H. pylori* SodB is associated with the cell surface [9]. SodB is the only identified Sod in *H. pylori* and has been shown to be critical for survival *in vivo* [10]. Also, *sodB* deficient mutants are more sensitive to O_2_ as well as exhibit a higher rate of spontaneous mutation [10], [11]. Interestingly, *H. pylori sodB* mutants have been shown to harbor more free iron within their cells than WT bacteria [11].

Globally, Sods are responsible for combating oxidative stress (both internal and external) by converting superoxide radicals into hydrogen peroxide and oxygen. Superoxide radicals are formed as a by-product of metabolism and, if left unchecked, can react with ferric iron (Fe^3+^) to form hydrogen peroxide, which in turn feeds the Fenton Reaction [12] and is detrimental to the cell. Sods prevent the interaction of iron and superoxide radicals as well as block the formation of hydroxyl radicals from hydrogen peroxide [12]. In this way, the role of Fur as the primary regulator of iron uptake and the role of SodB as the primary defense against superoxide radicals in *H. pylori* are linked. In keeping with this, *sodB* has been shown to be regulated by *apo*-Fur such that it is repressed under circumstances where iron is severely limited [1]. This regulation appears to be direct since Electrophoretic Mobility Shift Assays showed that Fur specifically binds to the *sodB* promoter in the absence of iron [1]. Herein we describe a series of experiments that define a single polymorphic nucleotide within the *H. pylori sodB* promoter that is important for *apo*-Fur dependent regulation. Moreover, we show that alterations in this single base result in strain specific responses to iron limitation.

## Materials and Methods

### Bacterial strains and growth

Strains and plasmids used in this study are listed in Table 1, and primer sequences are listed in Table 2. Strains of *H. pylori* were maintained as frozen stocks at −80°C in brain heart infusion broth (BD) supplemented with 10% fetal bovine serum (Gibco) and 20% glycerol (EMD Chemicals, Inc.). Bacterial strains were grown on horse blood agar (HBA) plates which contained 4% Columbia agar base (Neogen Corporation), 5% defibrinated horse blood (HemoStat Laboratories, Dixon, CA), 0.2% β-cyclodextrin (Sigma), 10 µg/ml vancomycin (Amresco), 5 µg/ml cefsulodin (Sigma), 2.5 U/ml polymyxin B (Sigma), 5 µg/ml trimethoprim (Sigma), and 8 µg/ml amphotericin B (Amresco). Liquid cultures of *H. pylori* were grown in brucella broth (Neogen Corporation) supplemented with 10% fetal bovine serum and 10 µg/ml vancomycin at 37°C with shaking at 100 rpm. As noted in Table 1, where appropriate, cultures and plates were supplemented with 8 µg/ml chloramphenicol (Cm) (EMD Chemicals, Inc.) and/or 25 µg/ml kanamycin (Kan) (Gibco). In addition, where detailed in the Materials and Methods, some HBA plates contained 5% sucrose (Suc) (Sigma). Both liquid and plate cultures were grown under microaerophilic conditions (5% O_2_, 10% CO_2_, and 85% N_2_) generated with an Anoxomat gas evacuation and replacement system (Spiral Biotech) in gas evacuation jars.

**Table 1:** Plasmids and strains used in this study.

Plasmid or strain	Description	Reference
Plasmids		
pTM117	Modified pHP666 to include *E. coli* origin and *rop* gene, *aphA-3* cassette (Kan^r^), multiple cloning site, and a promoterless *gfpmut3* gene	[18]
pDSM236	pTM117 *sodB* promoter::*gfpmut3*fusion	This study
pDSM368	pTM117 *pfr* promoter::*gfpmut3*fusion	[18]
pKSF-II	pEK::*kan-sacB*	[19], [20]
pDSM386	pGEM-T Easy::Δ*fur*	This study
pDSM387	pGEM-T Easy::Δ*fur*::*kan-sacB*	This study
pDSM469	pGEM-T Easy::Δ*sodB*	This study
pDSM475	pGEM-T Easy::Δ*sodB*::*kan-sacB*	This study
pDSM481	pGEM-T Easy::*sodB* C-5A	This study
pDSM429	pGEM-T Easy::26695 *fur*	This study
pDSM430	pET21A::26695 *fur*	This study
pKD4	*kan* template plasmid	[22]
pKD46	Red recombinase expression plasmid	[22]
*H. pylori* strains		
G27	WT *H. pylori*	[13]
DSM300	G27 Δ*fur*::*cat*, Cm^r^	[18]
26695	WT *H. pylori*	[14], [15]
DSM357	26695 Δ*fur*::*cat*, Cm^r^	This study
DSM238	G27 (pDSM236), Kan^r^	This study
DSM308	DSM300 (pDSM236), Kan^r^ Cm^r^	This study
DSM369	G27 (pDSM368), Kan^r^	[18]
DSM370	DSM300 (pDSM368), Kan^r^ Cm^r^	[18]
DSM391	G27 Δ*fur*::*kan-sacB*, Kan^r^ Suc^s^	This study
DSM403	G27, *fur* 26695, Suc^r^ Kan^s^	This study
DSM480	G27 Δ*sodB*::*kan-sacB*, Kan^r^ Suc^s^	This study
DSM491	G27 *sodB* C-5A, Suc^r^ Kan^s^	This study
J99	WT *H. pylori*	[16]
HPAG1	WT *H. pylori*	[17]
*E. coli* strains		
DSM328	K12 (pKD46), Amp^r^, Temp^s^	[22]
DSM355	K12 Δ*fur*, Kan^r^	This study
DSM326	BL21 DE3 Rosetta/pLysS, Cm^r^	This study
DSM365	BL21 DE3 Rosetta/pLysS Δ*fur*, Kan^r^, Cm^r^	This study
DSM431	BL21Δ*fur* (pDSM430) Amp^r^, Cm^r^, Kan^r^	This study

**Table 2:** Primers used in this study.

Primer^b^	Sequence (5′-3′)^a^	Reference
*sodB* promoter primers		
sodB-F1 (SacII)	CCGCGGCGCCATTGACCAATTTCAG	This study
sodB-R1 (BamHI)	GGATCCGCAACTCTCGTAATGTAAAC	This study
Screening and Sequencing primers		
gfp-1	AAGTCGTGCTGCTTCATGTG	[18]
aphA3-2	CGGTGATATTCTCATTTTAGCC	[18]
sacBSCN-F2	CGAATCGAATTCAGGAAC	This study
HpKanSacSCN-R	GGGAAGTTCTATGCTTATGG	This study
HpsodBSCN-R	GCTCGCTTCTTTAAACTCAACC	This study
Cloning primers		
FurCF (XbaI)	TCTAGAAAGGCTCACTCTACCCTATT	[18]
HpUKanSacR (XhoI, SmaI)	CTCTTGGCATTCTTTACACCACACCCCGGG **AGG** CTCGAGGCTGATATCTTCCTTATCCG	This study
HpDKanSacF (XhoI, SmaI)	CGGATAAGGAAGATATCAGCCTCGAG **CCT** CCCGGGGTGTGGTGTAAAGAATGCCAAGAG	This study
HpDKanSacR	CGCAGCGATAAAGGCGTGGTG	This study
FurCR (SalI)	GTCGACAAGACTTTCACCTGGAAACGC	[18]
USod-F	GCTTTATCGCCCACTTTCAAG	This study
USod-R	CCACAATAGCCGTAACGCTTACCCGGG **AGG** CTCGAGCATGTTTTCTCCTTGTGATTAG	This study
DSod-F	CTAATCACAAGGAGAAAACATGCTCGAG **CCT** CCCGGGTAAGCGTTACGGCTATTGTGG	This study
DSod-R	GGCATGGAATTGTCAATCC	This study
SodBMt-R	GATAGCCTTATTGTAATC	This study
SodBMt-F	GATTACAATAAGGCTATC	This study
HP_Fur_expression F2 (NdeI)	CATATGAAAAGATTAGAAACTTTGGAATCCATTTT	This study
HP_Fur_expression R2 (Xho1)	CTCGAGTTATTAACATTCACTCTCTTGG	This study
Red_EC_Fur_F	GAGCTGTAACTCTCGCTTTTCTTATTTCCCTTGCATGTGTAGGCTGGAGCTGCTTC	This study
Red_EC_Fur_R	TCATGTCTACGCCGTATTAATAGATAATGCCAATCACCATATGAATATCCTCCTTAGTTC	This study
RPA primers		
amiE-RPA-F	GGTTTGCCTGGGTTGGAT	[7]
amiE-RPA-R	GATTTTGCGGTATTTTG	[7]
pfr-RPA-F	GCGGCTGAAGAATACGAG	[18]
pfr-RPA-R	CTGATCAGCCAAATACAA	[18]
sodB-RPA-F	AAGCCCTGTAGCGTTTGATT	This study
sodB-RPA-R	CCCAATTCCAACCAGAGCCA	This study
fur RPA F	GAGCGCTTGAGGATGTCTATC	[18]
fur RPA R	GTGATCATGGTGTTCTTTAGC	[18]
EMSA primers		
G27 sodB EMSA-F	CTACAAAATTTGCATAACG	This study
26695 sodB EMSA-F	CCACAAAATTTGCATAAAG	This study
sodB EMSA-R	GCAACTCTCGTAATGTAAAC	This study
rpoB EMSA-F	CCAAAGAGGGTAAAGAGAGCG	This study
rpoB EMSA-R	CCTCTCCATCGCTTCTCTAAC	This study

a Restriction endonuclease sites are underlined, and linker bases are in bold type.

b Important restriction sites are included in parentheses.

*H. pylori* strains used in this study are all derivatives of G27 [13] and 26695 [14], [15], with the exception of WT *H. pylori* J99 [16] and HPAG1 [17]. A *fur* (HP1027) mutant of G27, DSM300, was utilized in this work and contains a deletion insertion of the *fur* coding sequence with the *cat* gene from *Campylobacter coli* conferring Cm resistance as previously described [18]. This ΔHP1027::*cat* construct was also naturally transformed into 26695 to create an analogous *fur* mutation in this strain background and is called DSM357. Exponential phase cultures were grown for 20 hrs, and stationary phase cultures were grown for 44 hrs.

### Creation of the *sodB* promoter fusion plasmid

A transcriptional fusion of the *sodB* (HP0389) promoter to the promoterless *gfpmut3* on the transcriptional reporter plasmid, pTM117, was constructed as previously described [18]. Briefly, the *sodB* promoter of WT G27 was PCR amplified using sodB-F1 and sodB-R1 primers, which incorporate SacII and BamHI restriction sites, respectively. The resulting PCR fragment was subcloned into pGEM-T Easy (Promega) and digested with SacII (New England Biolabs) and BamHI (Invitrogen). The resulting promoter fragment was then ligated into the appropriately digested pTM117 vector to create pDSM236. The fusion was confirmed by PCR amplification with sodB-F1 and gfp-1 [18] primers and by sequencing with the aphA3-2 primer [18]. pDSM236 was naturally transformed into WT G27 and DSM300, and transformants were selected on HBA plates containing 25 µg/ml Kan and 25 µg/ml Kan plus 8 µg/ml Cm, respectively. The WT strain bearing pDSM236 was designated DSM238, and DSM300 bearing pDSM236 was designated DSM308.

### GFP reporter assays

The ability of the *sodB* transcriptional fusion to drive the expression of GFP was assessed using flow cytometry as described previously [18]. Briefly, DSM238 was grown overnight in liquid culture with and without the iron chelator, 2,2′-dipyridyl (dpp) (Sigma) at a final concentration of 60 µM, and DSM308 was grown overnight in the absence of chelator. As a comparison, the previously characterized strains, DSM369 and DSM370, which bear *pfr* (*nonheme iron-containing ferritin*) transcriptional fusion plasmids in WT and Δ*fur* G27, respectively, were grown in the same manner [18]. Following overnight growth, 1.5 ml of each culture were pelleted and resuspended in 1 ml of sterile 1× phosphate-buffered saline. Bacterial clumps and culture debris were subsequently removed by passing the resuspended culture through a 1.2-µm Acrodisc PSF syringe filter (Pall). Flow cytometry analysis was performed using a Beckman Coulter Epics XL-MCL flow cytometer with a laser setting of 750 V for the *pfr* fusion construct and 900 V for the sodB fusion construct. 100,000 events were collected for each assay. WinList 3D, version 6.0 (Verity Software House) was used to analyze the flow cytometry data.

### Creation of the “Fur swap” Strain

To exchange the *fur* coding sequence, we first created a G27 strain containing the counter-selectable *kan*-*sacB* cassette previously described by Copass, et al [19]. This cassette contains the *sacB* gene from *Bacillus subtilis*, which confers Suc sensitivity and is expressed under the control of the *flaA* promoter of *H. pylori*, and the *aphA3* gene from *Campylobacter coli*, which confers Kan resistance. A 340 bp region upstream of the G27 *fur* coding sequence was PCR amplified using primers FurCF1 [18] and HpUKanSacR, and a 339 bp region downstream of the *fur* coding sequence was PCR amplified using primers HpDKanSacF and HpDKanSacR. HpUKanSacR and HpDKanSacF were designed to incorporate XhoI and SmaI restriction endonuclease sites. Each of these products were purified and mixed in a Splicing by Overlap Extension (SOE) PCR reaction using the FurCF1 and HpDkanSacR primers. The resulting 679 bp product was subcloned into pGEM-T Easy creating pDSM386. The *kan-sacB* cassette was liberated from pKSF-II [19], [20] by sequential double digestion with XhoI (New England Biolabs) and SmaI (New England Biolabs), and this fragment was ligated to the appropriately digested pDSM386 to create pDSM387. This plasmid was naturally transformed into WT G27, and transformants were selected on HBA plates containing Kan. Double crossover homologous recombination of pDSM387 with the WT chromosome results in the complete deletion of the *fur* (HP1027) coding sequence and replacement with the upstream *fur*-*kan-sacB*-downstream *fur* product. The resulting transformants were patched on 5% Suc HBA plates to ensure Suc sensitivity, and proper integration into the chromosome was confirmed by PCR with sacBSCN-F2 and HpKanSacSCN-R primers, which lie within the *sacB* gene and downstream of fur, respectively. One such transformant was named DSM391.

To create the “Fur swap” strain, a 923 bp product of the *H. pylori* 26695 genome was amplified using the FurCF and FurCR primers. This product, which includes the *fur* coding sequence and a portion of the upstream and downstream regions, was purified and naturally transformed into DSM391. Transformants were selected on 5% Suc HBA plates and patched onto Kan HBA plates to ensure Kan sensitivity. Double crossover homologous recombination resulted in the replacement of the *kan-sacB* cassette with the *fur* coding sequence of 26695, and this strain was named DSM403. Proper integration was confirmed by PCR with the FurCF and FurCR primers and by sequencing with the FurCR primer. DSM403 expresses 26695 *fur* from the native *fur* locus in a G27 strain background.

### Creation of a “−5 bp swap” mutation in the *sodB* promoter

The *sodB* promoter from G27 was sequenced using primers USod-F and DSod-R and compared to the known sequence of the *sodB* promoter from 26695 [14]. This comparison revealed a single base pair (bp) difference within the predicted Fur Box [1] at the −5 position relative to the start of transcription. The “−5 bp swap” mutation within the *sodB* promoter of G27 was created using SOE PCR and the *kan-sacB* cassette from pKSF-II. A 297 bp region upstream and a 329 bp region downstream of *sodB* were PCR amplified from G27 using primer pairs USod-F and USod-R and DSod-F and DSod-R, respectively. USod-R and DSod-F contain XhoI and SmaI restriction endonuclease sites to allow for the directional cloning of the *kan-sacB* fragment. The upstream and downstream products were purified and mixed in a SOE PCR reaction with the USod-F and DSod-R primers. The resulting 626 bp SOE PCR product was subcloned into pGEM-T Easy to create pDSM469. pDSM469 and pKSF-II were each sequentially double digested with XhoI and SmaI, and the resulting fragments were ligated to create pDSM475. pDSM475 was naturally transformed into WT G27, and transformants were selected on Kan and then patched to verify sucrose sensitivity. Double crossover homologous recombination of pDSM475 into the G27 chromosome results in the deletion of the *sodB* gene and replacement with the *kan-sacB* cassette. The resulting Kan resistant, sucrose sensitive strain, DSM480, was confirmed by PCR with sacBSCN-F2 and HpsodBSCN-R primers, the latter of which lies downstream of *sodB*.

The −5 bp in the G27 *sodB* promoter was mutated from a C to an A using SOE PCR. First, primers USod-F and SodBMt-R were used to PCR amplify upstream of the *sodB* promoter through to the −5 bp and incorporate the C-5A mutation. Second, primers DSod-R and SodBMt-F were used to PCR amplify from the −5 bp through to downstream of the sodB gene and to incorporate the C-5A mutation. These products were purified and combined in SOE PCR reaction using the USod-F and DSod-R primers. The resulting SOE PCR product was sublconed into pGEM-T Easy. The subcloned *sodB* −5 bp promoter mutation construct was designated pDSM481 and was confirmed by sequencing with the USod-F and DSod-R primers.

pDSM481 was naturally transformed into DSM480 to integrate the *sodB* −5 bp promoter mutation into the chromosome in place of the *kan-sacB* cassette. Transformants were selected as detailed above for the creation of DSM403. The resulting Suc resistant, Kan sensitive strain was named DSM491. Proper recombination was confirmed by PCR with the USod-F and DSod-R primers (yielding a 1,262 bp fragment) and by sequencing with both of those primers. DSM491 expresses *sodB* with the C-5A mutation from its native locus within the G27 chromosome.

### RNase protection assays (RPAs)

RPAs were utilized to characterize *apo*-Fur regulation of *sodB* in various strains of *H. pylori*. Two normal (iron replete) media cultures were started for each strain, one for exponential and one for stationary growth phase. Following overnight growth, one half of each exponential phase culture was removed for RNA isolation. To the remaining half of the iron-replete exponential phase cultures, 200 µM dpp (final concentration) was added to create an iron-depleted shock condition. Those cultures were grown for an additional hour prior to RNA isolation. The iron-replete stationary phase cultures were grown for an additional night, and on the following morning one half of the culture was removed for RNA isolation while the other was exposed to 200 µM dpp for an additional hour before RNA isolation. In addition, one culture for each strain was grown in iron limited media (60 µM dpp). After overnight growth, one-half of each culture was removed for RNA isolation in exponential phase. The remaining half of the iron-limited growth culture was allowed to grow overnight and was harvested the following morning for the stationary phase, iron-limited growth RNA samples. RNA was extracted as described previously [21]. RNase Protection Assays (RPAs) were performed as previously described [18] with 1.5 µg of RNA using *sodB*, *pfr*, *amiE*, and/or *fur* riboprobes that were generated using the primer pairs listed in Table 2. In brief, riboprobes were generated with 50µCi [^32^P]UTP (Perkin-Elmer) and a Maxiscript kit (Applied Biosystems). The RPA III kit (Applied Biosystems) was used for the RPA reactions that were resolved on 5% acrylamide-1× Tris-borate-EDTA-8M urea denaturing gels. The gels were exposed to phosphor screens, and the phosphor screens were scanned using a FLA-5100 multifunctional scanner (Fujifilm). Analyses and quantitation of the RPAs were performed using the Multi-Gauge software (version 3.0, Fujifilm). In all cases, three to four biological repeats of each experiment were performed.

### *H. pylori* Fur Expression and Purification

*H. pylori* 26695 Fur coding sequence was amplified using primers HP_Fur_expression F2 (NdeI) and HP_Fur_expression R2 (XhoI), and the PCR product was cloned into the pGEM-T easy vector (Promega) to create plasmid pDSM429. pDSM430 was created by proper digestion of pET21A (Novagen) and pDSM429 with NdeI and XhoI and ligation of the gel purified fragments. The Fur coding region in pDSM430 was sequenced to verify the construct. To prevent cross contamination of *H. pylori* recombinant Fur with *E. coli* endogenous Fur, an *E. coli* BL21 Rosetta *Δfur* strain was constructed using the Wanner method [22]. Briefly, the Kan resistance cassette was amplified from pKD4 [22] with primers Red_EC_Fur_F and Red_EC_Fur_R. This PCR product was introduced into arabinose induced *E. coli* K-12 carrying the pKD46 plasmid [22] to create DSM355. DSM365 was created by transduction of DSM326 with P1L4 grown on DSM355. Endogenous *E. coli* Fur deletion was verified by PCR. pDSM430 was introduced into DSM365 to create DSM431, which was used for rFur induction. DSM431 was grown to mid log in Luria-Bertani (EMD Chemicals) medium and then induced with 0.5 mM IPTG (isopropyl-D-thiogalactopyranoside) (Sigma) at 30°C for 3 h. The cells were disrupted using French press (Amicon) and crude extracts were prepared from the IPTG-induced cells by centrifugation (5,000 rpm for 30 minutes). Protein purification was performed by fast-protein liquid chromatography; the cytoplasmic protein was first passed through a HiTrap SP column for ion-exchange-based purification with a salt gradient of 25 mM to 500 mM NaCl (obtained by using buffer A [50 mM sodium phosphate, 25 mM NaCl, pH 8.0] and buffer B [25 mM sodium phosphate, 500 mM NaCl, pH 8.0]). Peak fractions containing Fur protein from the ion-exchange procedure were collected and further purified based on size exclusion by using a Sephacryl-200 column (buffer C [50 mM sodium phosphate, 200 mM NaCl, pH 8.0]). rFur was partially concentrated using an Amicon Ultra Centrifugal Filter Device (Millipore) to remove a portion of buffer C. Then an equal volume of EMSA binding buffer (BB) was added to the partially concentrated rFur with an additional 50% glycerol. rFur was further concentrated before being quantitated and stored at −20°C. The final concentration of the rFur stock was 2 mg/mL.

### Electrophoretic Mobility Shift Assays (EMSAs)

A 120 bp region of the *sodB* promoter (encompassing the Fur-box) [1] was PCR amplified using the following template and primer pairs: WT G27 and DSM491 (“−5 bp swap”) with G27 sodB EMSA-F and sodB EMSA-R and WT 26695 with 26695 sodB EMSA-F and sodB EMSA-R. To serve as a negative control in the EMSA studies, a 142 bp region of the *rpoB* promoter was amplified from WT G27 using the rpoB EMSA-F and rpoB EMSA-R primer pair. Each PCR product was acrylamide gel purified and resuspended in 1× Tris-EDTA (TE) buffer. 150 ng of each promoter region was end labeled with [^32^P] ATP (Perkin Elmer) using T4 polynucleotide kinase (New England Biolabs) as previously described [7]. The unincorporated nucleotide was removed using the MinElute Reaction Clean-up kit (Qiagen), and labeled promoter fragments were eluted twice with 10 µL EB, and 50 µL of *apo*-BB was added to the eluted product.

EMSAs were performed under *apo* (iron-free) conditions as previously described for WT 26695 *sodB* [1]. Briefly, 1 ng of labeled *sodB* or *rpoB* promoter was mixed with 5 µL of the following dilutions of the Fur stock: 1∶1,875, 1∶3,125, 1∶15,625, and 1∶78,125 and combined with 10 µL of 2× *apo*-BB (24% glycerol, 40 mM Tris, pH 8.0, 150 mM KCl, 2 mM DTT, 600 µg/mL bovine serum albumin, 200 µM EDTA, and 0.1 mg/mL sheared salmon sperm DNA). In addition, a no protein control reaction and a 100 ng cold (unlabeled) DNA competition reaction were performed. The cold competition reaction was performed with the highest concentration of Fur (1∶1,875). All reactions were allowed to incubate at 37°C for 30 min. After the incubation, the reactions were separated on a 5% polyacrylamide gel (5% 19∶1 acrylamide, 1× Tris Glycine EDTA (TGE) buffer, 2.5% glycerol) for 3 hours at 70 V in 1×TGE buffer. The gels were then exposed to phosphor screens and scanned on a Storm 860 scanner (GE Healthcare). Analysis was performed using ImageQuant version 5.2 software (Molecular Dynamics).

### Competition EMSA Studies

Competition studies were performed in a manner analogous to the EMSAs. Each labeled *sodB* promoter fragment was combined with the 1∶1,875 dilution of rFur, *apo*-BB, and either 5 ng, 10 ng, or 25 ng of cold (unlabeled) *sodB* promoter from each of the three respective strains. A no competitor control was included for each labeled *sodB* promoter fragment. In this manner, each labeled *sodB* fragment (WT G27, “−5 bp swap,” and WT 26695) competed for binding to Fur with its own unlabeled *sodB* fragment as well as to that of the other two strains. The incubations, electrophoresis, and analysis were performed as described for the EMSAs. Binding competition occurs as follows: 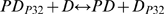, where P = Fur, D*_P32_* = labeled DNA, and D = cold competitor. Thus, if the competitor promoter fragment (D) can bind to Fur (P) with a higher affinity than the labeled promoter (D*_P32_*), then an increase in the amount of unbound, labeled promoter (D*_P32_*) would be seen. The percent of unbound, labeled *sodB* promoter was quantitated for each competition EMSA using densitometry as a means of comparing the relative affinity of each promoter fragment for Fur.

### Statistical Analysis

Two-tailed Student's *t*-tests were performed using Microsoft Office Excel 2003.

### Nucleotide sequence accession number

The nucleotide sequence of the *sodB* promoter is available from GenBank under accession number EU888136. The G27 *fur* sequence was previously reported [18] and is available as GenBank accession number EF537051.

## Results

### *apo*-Fur Regulation in *H. pylori*

**Figure 1:**
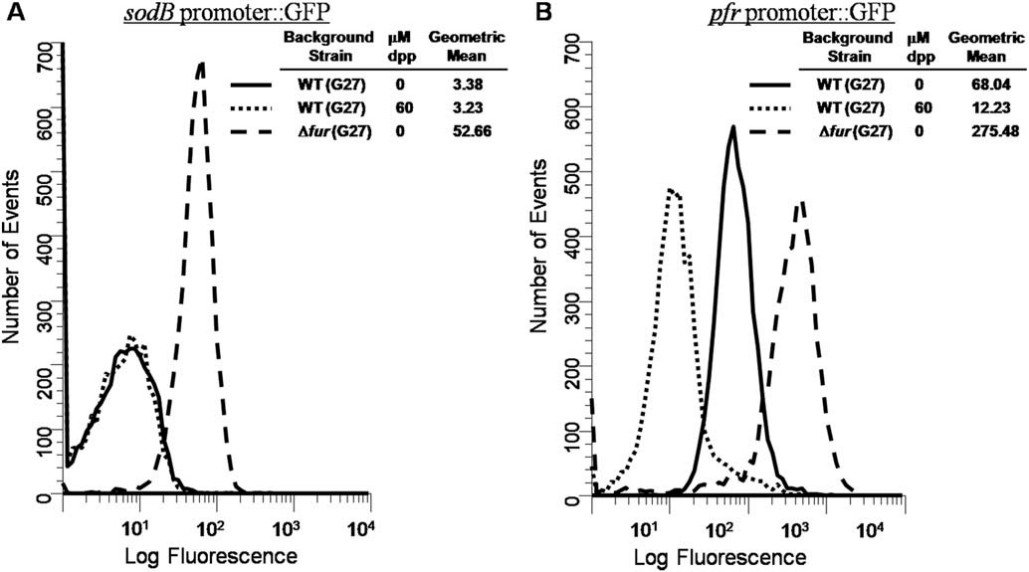
Flow Cytometry analysis of *sodB* and *pfr* GFP reporters. Strains bearing *sodB::gfpmut3* or *pfr::gfpmut3* promoter fusions were grown overnight in either iron replete or iron depleted media. Changes in fluorescence were analyzed as described in the Materials and Methods. Results for the *sodB* promoter fusions are displayed in Panel 1A, and results for the *pfr* promoter fusions are displayed in Panel 1B. For both A and B, solid lines indicate the plasmid in WT *H. pylori* G27 grown in iron replete conditions, dotted lines indicate the plasmid in WT bacteria grown in iron deplete conditions, and dashed lines indicate the plasmid in Δ*fur* bacteria grown in iron replete conditions. Fluorescence is measured in relative units, and the data are representative of multiple independent flow analyses.

In order to study *apo*-Fur dependent regulation in *H. pylori*, the *sodB* and *pfr* promoters from strain G27 were fused to the promoterless *gpfmut3* gene in pTM117. Currently, these promoters represent the only known targets of *apo*-Fur [1], [8]. Given this *apo*-regulation and since promoter activity can be measured by changes in fluorescence with our system, we expected to see a decrease in GFP fluorescence under iron limited conditions for both promoter fusions. However, as shown in Fig. 1A, the addition of iron chelator resulted in no change in the level of *sodB* expression. This is in contrast to *pfr*, where iron depletion resulted in strong repression of *pfr* expression (Fig. 1B). Both *sodB* and *pfr* were upregulated in a *fur* mutant (Fig. 1A and 1B) suggesting that both genes are repressed by Fur. However, the lack of responsiveness to iron chelation suggested that *sodB apo*-regulation is not as expected in G27.

**Figure 2:**
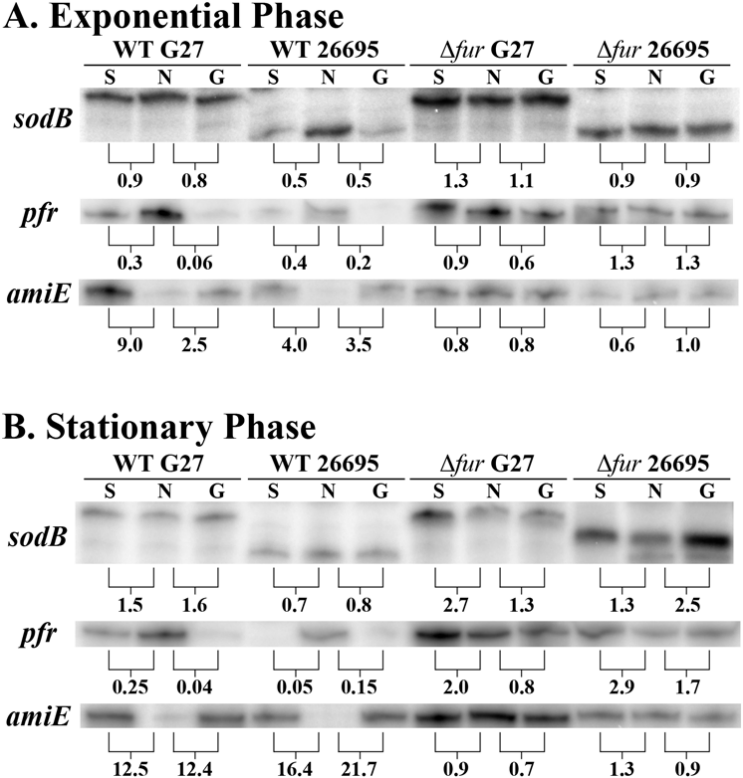
Direct Comparison of *sodB* Regulation in *H. pylori* Strains G27 and 26695. WT and Δ*fur* strains of G27 and 26695 were grown to exponential (A) and stationary (B) phase in iron replete and iron-limited (growth) media (60 µM dpp). After growth overnight, one-half of the exponential phase, iron replete culture was removed for RNA isolation. 200 µM dpp (final concentration) was added to create an iron-depletion shock condition to the remaining half of the iron replete cultures, and those cultures were grown for an additional hour prior to RNA isolation. The same procedure was applied the following day to the iron replete, stationary phase culture. After overnight growth, one-half of the iron-limited growth culture was removed for RNA isolation in exponential phase while the remaining half was allowed to grow into stationary phase, and RNA was isolated the following day. RNase Protection Assays (RPAs) were performed on RNA isolated from these strains using *sodB*, *pfr*, and *amiE* riboprobes. Data for Exponential phase cultures are shown in Panel A, and data for Stationary phase cultures are shown in Panel B. Fold-changes are indicated below each pair and were calculated by comparing either the relative amount of protected riboprobe in the iron-depletion shock environment (S) or the relative amount of protected riboprobe in the iron limited growth environment (G) to the iron replete lane (N). These data are representative of multiple independent experiments.

Since *apo*-Fur has been shown to have a lower affinity for the *sodB* promoter than the *pfr* promoter, and since the *gfpmut3* allele encodes a long-lived GFP variant [23], we reasoned that we might not be able to detect small changes in GFP expression under the control of the *sodB* promoter under iron limited conditions. Therefore, we performed RPAs to further investigate the discrepancy between our results and results previously reported for *sodB* regulation in strain 26695 [1]. Additionally, we considered the fact that strain specific differences might be responsible for the discrepancy. Therefore, RPAs using a *sodB* riboprobe were performed on RNA isolated from WT and Δ*fur* derivatives from both G27 and 26695. *pfr* and *amiE* (*aliphatic amidase*) riboprobes were also used as control *apo*-Fur and iron-bound Fur regulated target genes, respectively. Fig. 2A shows results for all three riboprobes using RNA isolated from exponential phase cultures. Again, we observed that for G27 the level of *sodB* expression did not change under iron-limited growth conditions (G) or under a harsher iron-depletion shock condition (S) that was added to ensure robust chelation as compared to normal (N) iron replete conditions.

Examination of *sodB* expression in 26695 revealed a smaller protected fragment than originally expected. However, sequence analysis revealed that the smaller fragment is due to a small region of mismatch between the *sodB* mRNA sequence in 26695 and the G27 template DNA used to generate the riboprobe. This mismatch causes a bubble of single stranded RNA to form and thus is subjected to RNase cleavage in the region of mismatch (data not shown). For WT 26695, a 2-fold decrease in *sodB* expression was achieved under both iron-limited growth and iron-depletion shock conditions, which agrees with the previous report [1]. This change is Fur-dependent as there is no change in *sodB* expression under either iron depletion condition in the absence of *fur*.

Since it has been shown that growth phase strongly affects gene expression in *H. pylori* [21], we performed similar experiments on RNA harvested from stationary phase cultures. As shown in Fig. 2B, we obtained identical results with the exception that the fold decrease seen in *sodB* expression was less pronounced in 26695 in this growth phase. Again, there was no decrease in *sodB* expression in G27, indicating that growth phase is not responsible for the differences in our results. Moreover, the difference in *sodB* regulation between the two strains is not the result of a generalized difference in *apo*-Fur regulation between G27 and 26695 since the appropriate decrease in *pfr* expression [8] was observed in both strains under iron-limited growth and iron-depletion shock conditions (Fig 2A and 2B). Furthermore, iron-bound Fur regulation of *amiE* was as expected [24] for both G27 and 26695; *amiE* expression was increased under both iron limited conditions (Fig. 2A and 2B). Taken in total, these data indicate that *apo*-Fur regulation of *sodB* is altered in G27 as compared to 26695.

### Analysis of the role an amino acid (AA) difference in Fur plays in *sodB* regulation

**Figure 3:**
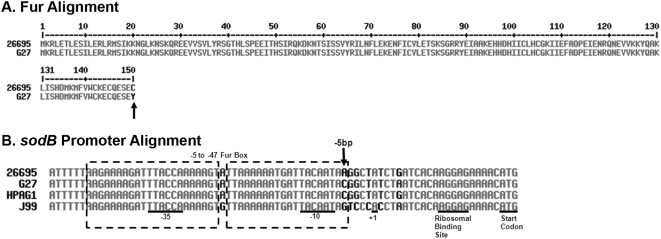
Alignments of Fur and of the *sodB* promoters. Panel A contains the alignment of the predicted Fur amino acid sequences of G27 and 26695. As indicated by an arrow, amino acid 150 is different between the two strains. Panel B contains the *sodB* promoter alignment from G27, 26695, J99, and HPAG1 with essential promoter elements indicated. The predicted Fur Box ranges from bases −5 to −47 and is indicated by the dashed box [1]. The −5 bp difference between the strains is indicated with an arrow in Panel B. Alignments for both panels were constructed using MultAlin software [37].

**Figure 4:**
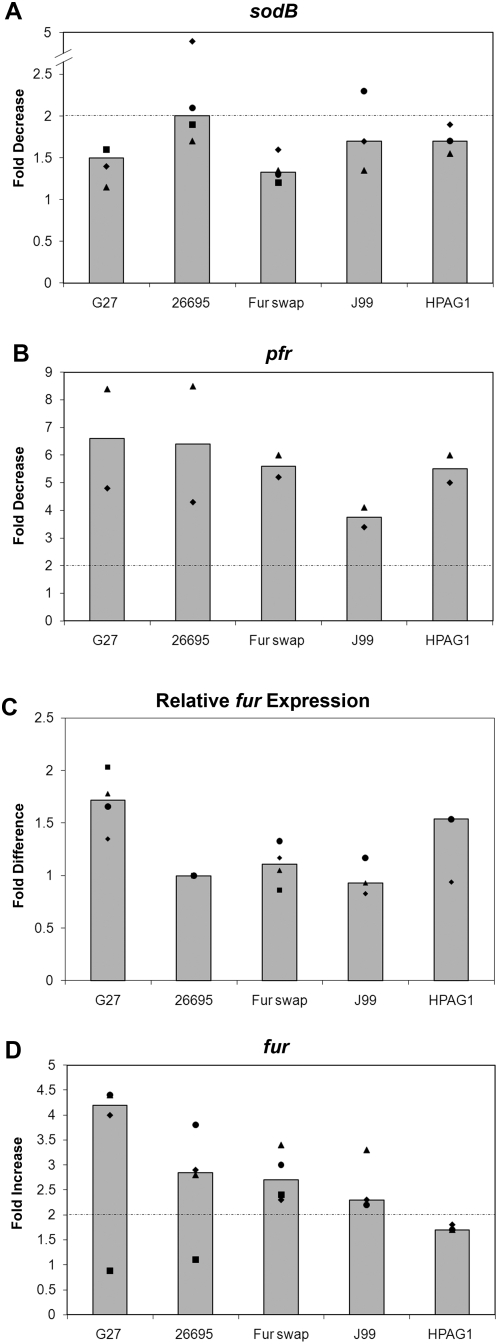
Strain specific differences in *sodB* regulation. Various *H. pylori* strains were grown to exponential phase as described in the Materials and Methods, and RNA was isolated from iron replete and iron-depleted shock conditions. RPAs were performed using *sodB*, *pfr*, and *fur* riboprobes and results are displayed in Panels A, B, and D, respectively. Basal levels of *fur* expression relative to the level of expression in 26695 are depicted in Panel C. Fold decrease in expression for *sodB* and *pfr*, fold increase for *fur*, and relative levels of basal *fur* expression are plotted as single points for each strain with squares, diamonds, triangles, and circles. Each shape represents a biologically independent set of RNA. Median fold change is represented as a bar for each strain. The dotted-dashed line represents the 2-fold significance cut-off in Panels A, B, and D. In Panel A only, the triangles represent the average of two technical repeats on that independent set of RNA.

Given the difference in *sodB* regulation between the two strains, we reasoned that either a difference in Fur or a difference in *sodB* between the two strains was likely to be responsible for the change. We therefore aligned the predicted Fur amino acid sequence from G27 and 26695 to determine if there were any obvious differences between the two strains that might account for the differences in *sodB* regulation. As shown in Fig. 3A, the last AA was found to differ between the strains. In G27 AA 150 is a Tyr while in 26695 it is a Cys. To determine if this AA difference had any role in Fur-dependent regulation of *sodB*, a “Fur swap” strain was created, which completely replaced the G27 *fur* coding sequence with the coding sequence from 26695. RPAs were then conducted on RNA harvested from WT G27, WT 26695, and the “Fur swap” strain. Results are shown in Fig. 4. In order to show the reproducibility of the data, RPA data is represented in a graphical format. In this manner the fold change for each strain and biological repeat is displayed as a point on the graph. Additionally, the median fold change is depicted as a bar to allow for easy comparison between the strains. Because the decrease in *sodB* expression in 26695 is most pronounced in exponential phase, only results of RPAs performed using exponential phase RNA are shown. Expressing 26695 Fur in G27 (the “Fur swap” strain) did not restore *apo*-Fur *sodB* regulation in G27 under either iron-limited growth or iron-depletion shock conditions (Fig. 4A and data not shown). However, *apo*-Fur regulation of *pfr* was as expected in all three strains (Fig. 4B and data not shown) [8]. Because the trends of the growth data for both the *sodB* and *pfr* RPA data were similar to the shock, the growth data has not been shown.

While the AA difference in Fur was apparently not responsible for the difference in *sodB* regulation, we wondered if the levels of *fur* expression were similar between the different strains. To test this, RPAs were performed on RNA isolated from all three strains using a *fur* riboprobe. The basal level of *fur* expression in each strain was then compared to that of WT 26695 as shown in Fig. 4C. While the level of *fur* expression in the G27 strain was slightly higher than in 26695, no substantial differences in *fur* expression were found between the strains.

As Fur has been shown to be autoregulatory, repressing its own expression in the presence of iron [25], [26], we also compared Fur autoregulation between G27, 26695, and the “Fur swap” strain. *fur* RPAs were performed on RNA isolated from each strain, and an increase in *fur* expression was seen for G27, 26695, and the “Fur swap” strain under iron-depletion shock conditions while little to no increase was seen under iron-limited growth conditions (Fig. 4D and data not shown). This data shows that Fur autoregulation is consistent in each strain and further supports the notion that the AA difference in Fur is not responsible for the difference in *sodB* regulation between G27 and 26695.

### RPA determination of the role the −5 bp of the *sodB* promoter plays in *sodB* regulation

**Figure 5:**
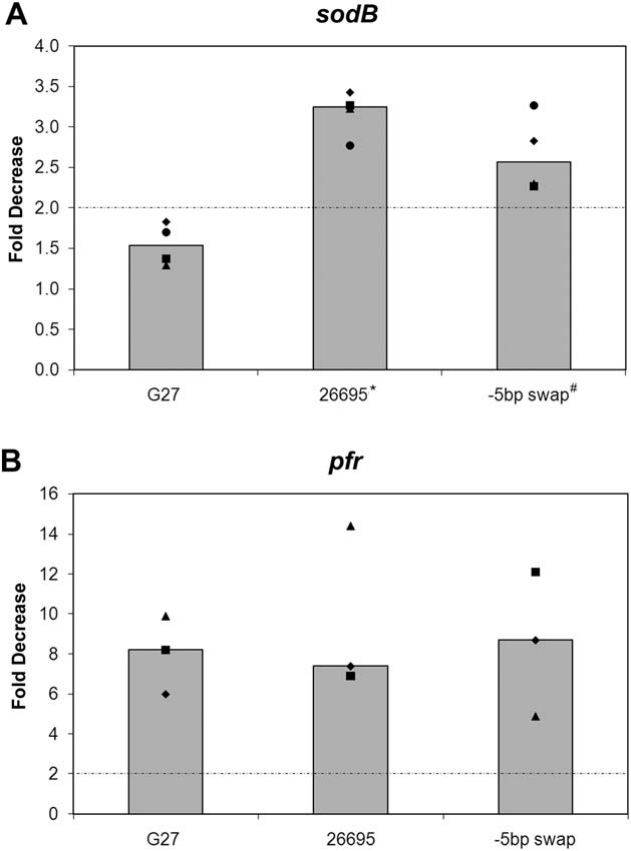
Role of the −5 bp in *sodB* regulation. WT G27, WT 26695, and the “−5 bp swap” strain were grown as described in the Materials and Methods, and RNA was isolated under iron replete and iron-depletion shock conditions. RPAs were performed on RNA isolated from 4 biologically independent experiments using *sodB* and *pfr* riboprobes. Data from *sodB* RPAs are presented in Panel A, and data from *pfr* RPAs are presented in Panel B. Each square, diamond, triangle, and circle represent the average fold decrease calculated from three technical repeats with each independent set of RNA for each strain and growth condition combination. Median fold decrease is represented as a bar for each combination, and the dotted-dashed line represents the 2-fold significance cut-off. ^*^p-value of 0.0001. ^#^p-value of 0.006.

Since the difference in *sodB* regulation between G27 and 26695 appeared not to be related to the difference in the Fur coding sequence, we next considered that there might be differences in the *sodB* promoter between the strains that could account for the discrepancy in regulation. Therefore, we sequenced the *sodB* promoter from G27 and compared it to the known *sodB* promoter sequence from 26695 [14]. As shown in Fig. 3B, a single base change was evident in the Fur Box. Previous DNA Footprint analysis showed that Fur protects a region that extends from −5 bp to −47 bp within the *sodB* promoter [1]. At the −5 bp, G27 encodes a C while 26695 encodes an A. To determine if this nucleotide difference was important for *sodB* regulation, a “−5 bp swap” strain was engineered such that the G27 promoter would encode an A at the −5 bp position. RPAs were then conducted on RNA isolated from the “−5 bp swap” strain along with WT G27 and WT 26695, and results are shown in Fig. 5. While *sodB* expression remained unchanged in G27 under iron depletion shock conditions, a two-fold decrease in *sodB* expression was observed in the “−5 bp swap” strain (Fig. 5A). The difference in fold decrease between G27 and the “−5 bp swap” was statistically significant with a p-value of 0.006, as was the difference between G27 and 26695 with a p-value of 0.0001. While the fold decrease in *sodB* expression in the “−5 bp swap” strain under iron-limited growth conditions did not reach 2-fold, it was consistently higher than its G27 counterpart (data not shown). *apo*-Fur regulation of *pfr* in each of these strains was similar and as expected [8] (Fig. 5B and data not shown). These data suggest that a single nucleotide difference within the *sodB* promoter is at least partially responsible for the difference in regulation of this gene between G27 and 26695.

### Comparison of *sodB* regulation in various strains of *H. pylori*

Given the differences in *sodB* regulation in G27 and 26695, we wondered if other *H. pylori* strains exhibited *apo*-Fur regulation similar to G27 or 26695. Therefore, we also examined J99 and HPAG1. Analysis of the *sodB* promoter sequences of these two additional strains showed that at the −5 bp HPAG1 encodes a C similar to G27, and J99 encodes a G that is different from all other strains (Fig. 3B). Given that the A at the −5 bp seems to be crucial for *apo*-Fur regulation of *sodB*, we predicted that these strains would show Fur regulation of *sodB* similar to what was seen with G27. To test this, RPAs were performed on RNA isolated from J99 and HPAG1. As shown in Fig. 4, neither J99 nor HPAG1 displays the expected decrease in *sodB* expression [1]; both behave similarly to G27 (Fig. 4A). However, *pfr* expression (Fig. 4B), basal levels of *fur* expression (Fig. 4C), and *fur* autoregulation (Fig. 4D) are preserved in J99 and HPAG1. Taken together, these data suggest that natural polymorphisms found at the −5 bp of the *sodB* promoter in different *H. pylori* strains affect the regulation of *sodB* by *apo*-Fur.

### *In vitro* binding of Fur to different *sodB* promoters

**Figure 6:**
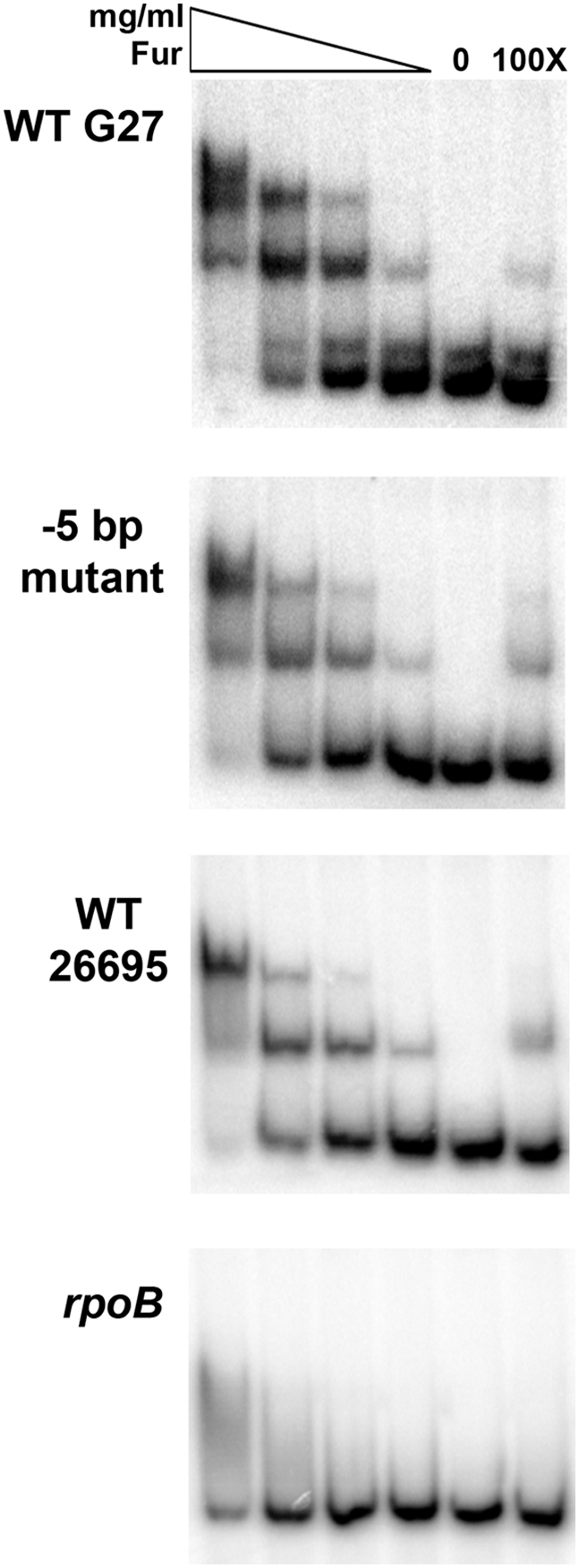
Fur binding to the *sodB* promoters. EMSAs were performed by incubating various concentrations of purified Fur with radiolabeled fragments of the WT G27, “−5 bp swap,” and WT 26695 *sodB* promoters as well as the negative control promoter, *rpoB*, as detailed in the Materials and Methods. In the first four lanes, the Fur concentrations are indicated by the triangle from highest to lowest and range from 1.07 µg/mL to 0.026 µg/mL. A no protein control for each promoter is found in the fifth lanes. The last lane shows the 100× cold (unlabeled) competition control for each promoter fragment, which were each performed with the highest concentration of Fur (1.07 µg/mL). Fur exhibits specific interaction with each of the *sodB* promoters, and no interaction with the *rpoB* promoter except for very little non-specific binding at the highest Fur concentration. These data are representative of multiple independent EMSA experiments.

Given that the −5 bp in the *sodB* promoter appears to play some role in the *apo*-Fur regulation of *sodB*, we next investigated the direct interaction of *apo*-Fur with the various *sodB* promoters. To assay the binding of *apo*-Fur, we performed Electrophoretic Mobility Shift Assays (EMSAs) and competition studies for each *sodB* promoter (WT G27, “−5 bp swap,” and WT 26695) using purified Fur under *apo* reaction conditions [1]. As shown in Fig. 6, Fur binds to and retards the mobility of each of the three *sodB* promoters, but not the control *rpoB* promoter. Moreover, the addition of homologous unlabeled *sodB* promoter DNA was able to compete for Fur binding with each *sodB* promoter thus confirming specific interaction between Fur and the *sodB* promoters (Fig. 6).

**Figure 7:**
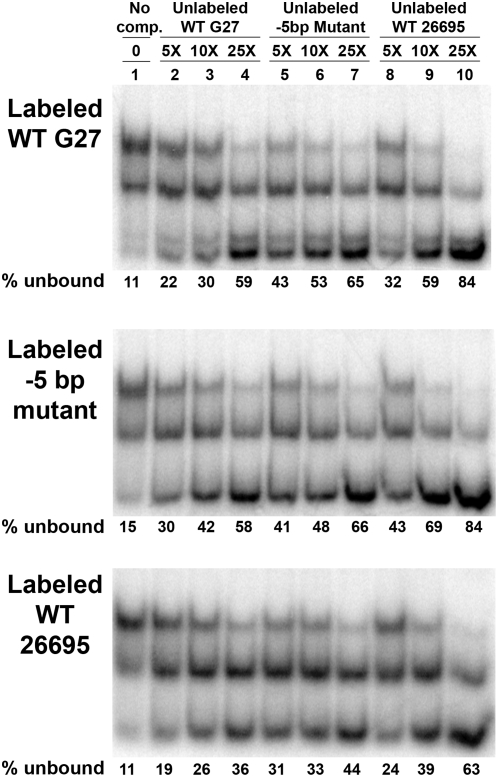
Competitive Binding Studies. To assess the relative affinity of Fur for each of the *sodB* promoter fragments (WT G27, “−5 bp swap,” and WT 26695), Fur was incubated with each radiolabeled promoter and 5×, 10×, or 25× the amount of homologous or heterologous unlabeled *sodB* promoter fragments as described in the Materials and Methods. For each labeled promoter, lane one contains a no competition control. Lanes two to four, five to seven, and eight to ten contain the competition EMSAs with unlabeled WT G27, “−5 bp swap,” and WT 26695 *sodB* fragments, respectively. The percent of labeled promoter that is outcompeted and remains unbound in each lane is given below each image. These data are representative of multiple independent experiments.

Because *apo*-Fur was able to bind to and shift each of the three *sodB* promoter fragments and because our expression data showed that the −5 bp was important for regulation, we reasoned that the various promoter fragments should show differences in their affinity for Fur. To test this, each labeled *sodB* promoter fragment was competed with varying concentrations of its own (homologous) unlabeled promoter fragment as well as with each of the other unlabeled promoter fragments. The success of the competition was then measured by quantitating the percent of unbound probe resulting from each competition reaction such that 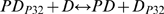, where P = Fur, D*_P32_* = labeled DNA, and D = cold competitor. As shown in Fig. 7, the various promoter fragments showed differences in affinity such that 26695≥−5 bp>G27. In all cases, the 26695 and −5 bp promoter were better able to compete for Fur binding as the largest percentages of unbound labeled promoter fragment are observed with these two promoters in comparison to the WT G27 *sodB* promoter. Taken together with the expression data, these data indicate that the −5 bp is important for Fur interaction at the *sodB* promoter.

## Discussion

Given how pleomorphic *H. pylori* is, it is not surprising that genes may be regulated differently in different strains. Indeed, there have been several instances of this reported in the literature in recent years involving acid-response and CrdRS [27], *vacA* regulation [28], virulence gene regulation *in vivo* [29], and *cagA* and *vacA* expression in response to salt [30]. In addition, a single nucleotide polymorphism upstream of the Fur-box was found to alter Fur regulation of *IrgA* in two different strains of *E. coli* [31] indicating that there may be more to Fur regulation in other organisms than just binding at the recognition sequence. This study adds to that body of knowledge and is the first to explore the differences in Fur regulation among different strains of *H. pylori*.

*apo*-Fur regulation remains a unique form of Fur regulation found only in *H. pylori*. Additionally, our understanding of this type of regulation is currently limited as only two *apo*-Fur repressed genes, *sodB* [1] and *pfr* [8], have been characterized. Here we present evidence that *H. pylori* shows strain specific differences in *sodB apo*-regulation that are partially controlled by a natural polymorphism found at the −5 bp of the *sodB* promoter. Alteration of this single nucleotide in the G27 promoter to resemble the residue found in 26695 resulted in alteration of G27 *sodB* regulation that mimicked regulation seen in 26695. Based on this observation, we accurately predicted that two other commonly used strains of *H. pylori*, J99 and HPAG1, would show altered *sodB* regulation since they each encode a different nucleotide at the −5 position within the *sodB* promoter.

The importance of the −5 bp within the *sodB* promoter is further supported by our EMSA competition data. At low concentrations of competitor DNA, the “−5 bp swap” promoter is able to bind to *apo*-Fur with an affinity similar to WT 26695 while WT G27 exhibits weaker binding. At higher concentrations of competitor, the affinity of the “−5 bp swap” promoter for *apo*-Fur is still greater than WT G27 but slightly less than WT 26695. Thus, it appears that strain specific regulation of *sodB* is due to differences in the affinity of Fur for the various promoters and that natural polymorphisms at the −5 bp are largely responsible for this differential regulation.

The significance of the *sodB* polymorphism in *H. pylori* fitness, especially *in vivo*, is currently unclear. However, the affinity of *apo*-Fur for the *sodB* promoter in 26695 was reported to be relatively weak (K*_d_* = 260 nM) [1], and based upon our competition data it is likely even weaker in G27. As Ernst, et al. suggested, a weak affinity between *apo*-Fur and the *sodB* promoter makes physiological sense, as SodB is the only defense *H. pylori* has against superoxide radical damage [1], [10]. Therefore, it would be ill-advised to repress *sodB* under conditions where any iron is still available, since iron catalyzed oxidative damage could still be possible [1]. In keeping with this, some strains of *H. pylori* may have evolved to either inactivate *apo*-Fur regulation of *sodB*, or to weaken repression by decreasing the Fur/*sodB* binding affinity. Also of note, as shown in Fig. 2, in the absence of Fur, iron chelation results in slight increases in *sodB* (and *pfr*) perhaps suggesting the presence of additional regulatory proteins that ensure proper expression of this critical factor.

Furthermore, it is interesting to speculate that strains, which possess sequences similar to 26695, might actually show decreased *in vivo* fitness due to decreased expression of *sodB* in the iron limited environment of the stomach. Analysis of the *sodB* promoter sequence in the efficient gerbil colonizing strain B128 (isolate 7.13) [32] revealed that B128, similar to G27, encodes a C at the −5 bp (data not shown). Therefore, studies could potentially be designed with this strain that would allow for the determination of whether direct *apo*-Fur regulation of *sodB* provides a competitive advantage to *H. pylori in vivo*.

Currently, little is understood about the sequences recognized by *H. pylori* Fur that dictate binding of the protein at target promoters. This is true of both iron-bound and *apo* forms of Fur. In *E. coli*, Fur binding has been shown to involve recognition of a well-conserved consensus sequence called a Fur Box. This Fur Box consists of two 9 bp inverted repeat sequences separated by a single A nucleotide to create a 19 bp palindromic sequence as follows: GATAATGATAATCATTATC [33]. This sequence can also be interpreted as a series of three hexameric repeats of NATA/TAT [34]. However, in *H. pylori* this *E. coli* Fur Box is not conserved, and consensus is currently ill-defined. For iron-bound Fur regulation, the binding sequence occurs in A/T-rich regions in the target promoter oftentimes with repeats of AAT [8], [24], [25], [35], [36]. There is no defined consensus sequence for *apo*-Fur binding given that the two promoters of the known *apo*-Fur regulated genes, *pfr* and *sodB*, share only minimal homology [1], [8]. In an organism that has about 60% A/T residues in its genome, a Fur Box consensus sequence that is comprised of mainly these two nucleotides does not seem to be an ideal approach for Fur regulation. Rather, in *H. pylori* it is perhaps more plausible that both iron-bound and *apo*-Fur recognize unique DNA structures that are required for proper regulation of their target genes. The work presented here is the first to define a residue that is important for *apo*-Fur binding to the *sodB* target promoter. Future work from our group will focus on elucidating binding residues important for both iron-bound and *apo*-Fur regulation with the hope that continued exploration of Fur regulation will provide greater understanding into the complexity of gene regulation in this important human pathogen.
